# The economic burden of pulmonary arterial hypertension in Spain

**DOI:** 10.1186/s12890-022-01906-2

**Published:** 2022-03-26

**Authors:** Néboa Zozaya, Fernando Abdalla, Ignacio Casado Moreno, Carlos Crespo-Diz, Ana M. Ramírez Gallardo, Joaquín Rueda Soriano, Macarena Alcalá Galán, Álvaro Hidalgo-Vega

**Affiliations:** 1 Department of Health Economics, Weber, Calle Moreto, 17, 5º Dcha., 28014 Madrid, Spain; 2 Department of Quantitative Methods in Economics and Management, University Las Palmas de Gran Canaria, Las Palmas, Spain; 3 Pneumology Unit, University Hospital Virgen de las Nieves, Granada, Spain; 4 Pharmacy Department, Complexo Hospitalario Universitario de Pontevedra, Instituto de Investigación Sanitaria Galicia Sur (IISGS), Pontevedra, Spain; 5 Pulmonary Hypertension Unit, Hospital Clínic, Barcelona, Spain; 6 Department of Cardiology, Hospital Universitari i Politècnic La Fe, Instituto de Investigación Sanitaria La Fe, CIBERCV, Valencia, Spain; 7 Market Access Department, Janssen, Madrid, Spain; 8 Weber Foundation, Madrid, Spain; 9 Department of Economic Analysis and Finances, University of Castilla-La Mancha, Toledo, Spain

**Keywords:** Economic impact, Burden, Social perspective, PAH, Pulmonary arterial hypertension

## Abstract

**Background:**

Pulmonary Arterial Hypertension (PAH) is a rare, debilitating, and potentially fatal disease. This study aims to quantify the economic burden of PAH in Spain.

**Methods:**

The study was conducted from a societal perspective, including direct and indirect costs associated with incident and prevalent patients. Average annual costs per patient were estimated by multiplying the number of resources consumed by their unit cost, differentiating the functional class (FC) of the patient. Total annual costs per FC were also calculated, taking the 2020 prevalence and incidence ranges into account. An expert committee validated the information on resource consumption and provided primary information on pharmacological consumption. Unit costs were estimated using official tariffs and salaries in Spain. A deterministic sensitivity analysis was conducted to test the uncertainty of the model.

**Results:**

The average annual total cost was estimated at €98,839 per prevalent patient (FC I-II: €65,233; FC III: €103,736; FC IV: €208,821), being €42,110 for incident patients (FC I-II: €25,666; FC III: €44,667; FC IV: €95,188). The total annual cost of PAH in Spain, taking into account a prevalence between 16.0 and 25.9 cases per million adult inhabitants (FC I-II 31.8%; FC III 61.3%; FC IV 6.9%) and an incidence of 3.7, was estimated at €67,891,405 to €106,131,626, depending on the prevalence considered. Direct healthcare costs accounted for 64% of the total cost, followed by indirect costs (24%), and direct non-healthcare costs (12%). The total costs associated with patients in FC I-II ranged between €14,161,651 and €22,193,954, while for patients in FC III costs ranged between €43,763,019 and €68,391,651, and for patients in FC IV between €9,966,735 and €15,546,021. In global terms, patients with the worst functional status (FC IV) account for only 6.9% of the adults suffering from PAH in Spain, but are responsible for 14.7% of the total costs.

**Conclusions:**

PAH places a considerable economic burden on patients and their families, the healthcare system, and society as a whole. Efforts must be made to improve the health and management of these patients since the early stages of the disease.

**Supplementary Information:**

The online version contains supplementary material available at 10.1186/s12890-022-01906-2.

## Background

Pulmonary arterial hypertension (PAH) is a rare, progressive, and life-threatening disease, defined by a chronic elevation of the pulmonary arterial pressure and pulmonary vascular resistance, which may lead to severe disability and even death if untreated [1, 2]. The symptoms of PAH include shortness of breath during activity, dizziness, fatigue, chest pain, fainting, palpitations, and peripheral oedema [3, 4]. Moreover, mental disorders like anxiety and depression are also reported to be frequent among patients with PAH [5–7]. The symptoms of the disease affect the patient’s physical mobility and emotional state, which adversely affect their health-related quality of life. In addition to the clinical impact, PAH imposes a substantial financial burden on both patients and their families, healthcare systems, and society as a whole [8].

The disease is significantly influenced by its functional class (FC). The FC scoring system categorises patients with pulmonary hypertension according to their perceived physical activity limitation, between FC I representing no limitations and FC IV constituting very high impediments, including the onset of symptoms at rest. Mortality rates after one year of diagnosis are 2.8% for FC I-II patients, 9.9% for FC III patients, and 21.2% for FC IV patients [9].

PAH belongs to group 1 of pulmonary hypertension, encompassing idiopathic PAH, hereditary PAH, drug induced PAH, and PAH associated with other diseases (e.g., connective tissue diseases, congenital cardiopathies, portal hypertension, HIV and Schistosomiasis, among others). These share similar clinical, physiological, and pathological aspects, and usually respond to the same treatments [10].

There is currently no cure for PAH. However, new therapeutic approaches to the disease have been developed within the last decade, aiming at relieving symptoms, improving quality of life, slowing disease progression, and prolonging life [11]. Pharmacological treatment for PAH targets three fundamental signalling pathways in the control of pulmonary vasomotor tone and vascular cell proliferation: the prostacyclin pathway (iloprost, treprostinil, epoprostenol, or selexipag), the endothelin pathway (ambrisentan, bosentan, or macitentan), and the nitric oxide pathway (sildenafil, tadalafil, or riociguat) [1, 12, 13].

Some of the patients go through hospitalization periods due to the disease or comorbidity-related issues. Moreover, lung and/or heart transplants should be considered as an option when other treatments fail and in those cases in which patients have not been able to progress to milder stages of the disease (FC I-II) [10]. In addition, the management of patients with PAH includes other measures, such as frequent medical visits, specific tests, rehabilitation, physical activities, psychological support, programs to support treatment adherence, and other types of therapy (e.g., diuretic treatments, anticoagulants, or the treatment for anaemia, among others) [10].

From a social point of view, PAH is a highly limiting disease, which requires a high amount of formal and informal care. Since the average age of PAH diagnosis is approximately 45 years old and in many cases the disease remains undiagnosed [12, 14], PAH may also have a high impact on the employment situation of patients, which could contribute to an increase of the total economic burden [15–18].

One of the tools used to inform health decision-making is disease burden studies, which allow understanding the consequences of the disease and estimating the necessary resources for its management. This can be especially relevant in the field of rare diseases, where there is less knowledge about the pathology and its potential associated costs, both for the patient and his/her family environment, as well as for the health system and society at large.

Previous studies have shown that PAH has a high psychological impact, requires formal and informal care, and represents a significant economic burden for health systems [8, 19–30]. However, to date, no study has estimated the indirect costs derived from the disease, nor studied its economic burden in Spain.

The objective of this study was to estimate the economic burden of PAH in Spain, from a social perspective. Specifically, we analysed the economic and social impact of the disease according to FC.

## Methods

### Design

A bottom-up prevalence approach was used to estimate the economic burden of PAH in Spain for the year 2020. Incident and prevalent patients with Group 1 PAH (all subtypes) were included, differentiated by their specific functional class (FC I-II, FC III, or FC IV).

The study was performed from a social perspective, including direct healthcare costs (DHC) for the National Health System (NHS) associated with the management of patients with PAH, as well as direct non-healthcare costs (DNHC), and indirect costs (IC) associated with the loss of labour productivity due to the disease of patients with PAH. This has been done according to previously published methodological [31, 32] and applied economic burden studies in Spain [33, 34] and elsewhere [35–37]. Specifically, we have included the following items under each cost category: (i) DHC: diagnosis, medical visits, tests, hospitalisations, transplant and drugs; (ii) DNHC: formal care, informal care, supportive therapies, medical devices; (iii) IC: early retirement, permanent leave, temporary leave, working hours reduction, working days missed, work time lost due to visits/tests and trips.

### Data sources

Data on epidemiology and resource consumption of patients with PAH were obtained from a literature review, including national and international references. Databases consulted were Medline/Pubmed and grey literature, as well as official databases. Spanish studies, and those with data disaggregated by FC were prioritised.

A multidisciplinary group of nationally recognised experts validated the data. Moreover, experts analysed the suitability of adapting data from other countries to the Spanish context and confirmed the validity of specific values deriving from variables for which a high data heterogeneity resulted from the literature search. In addition, two experts provided anonymised primary data on PAH patient medication utilisation from their own centres.

The expert group consisted of a pneumologist from one of the main public hospitals in the province of Granada (south region of Spain), a cardiologist from a Reference Centre (a unit which meets specific quality criteria on the management of a specific disease, such as knowledge, volume of patients treated, history, staff, results, information systems, etc.) in the treatment of PAH (Valencia, west of Spain), a pharmacist from the Regional Healthcare Management Area of Galicia (northwest of Spain), and a nurse, specialist in PAH in a Reference Centre in the treatment of PAH (Catalonia, northeast of Spain). All experts have a broad experience in the clinical management of PAH, as well as in research related activities, having participated as authors or co-authors in national and international publications. The number and profile of experts included are in line with the methodologies used in previous economic burden studies undertaken in Spain [33, 38].

Most of the data on DNHC and IC of patients with PAH came from a recent survey which was carried out by *Hipertensión Pulmonar España Organización de Pacientes* (Spanish Pulmonary Hypertension Patient Organisation, HPE-ORG) [39]. The anonymised database that originated from this survey was shared with the authors of this study. For data that was not available in that survey, information published in similar studies was considered.

The unit costs of the different resources analysed were estimated through rates and salaries in Spain, that had been published in the different regional regulations or in official national sources [40–65].

### Resource consumption

#### Direct healthcare costs

DHC included diagnosis and healthcare management. We assessed the mean number of annual medical visits, emergency department visits, tests, hospitalisations, transplants, and PAH-related medication consumption during diagnosis and follow-up, per patient and by FC (Table 1).

**Table 1:** Non-pharmaceutical DHC resource consumption per patient (diagnosis and follow-up by FC) and unit costs

	Resource consumption (n)						Unit cost (€)	
	Diagnosis	Ref	1 year follow-up			Ref	EUR	Ref
			FC I-II	FC III	FC IV			
Medical visits								
General practitioner	5.0	Experts	2.6	3.8	5.9	[66]	€51.88–€69.24	[40–55]
Nurse	–		2.8	5.5	8.8	[66]	€25.89–€27.70	[40–45, 47–50, 52–54, 57, 58, 60, 64, 65]
Specialised care	4.0		2.8	4.2	7.1	[66]	€89.15–€158.80	[40–43, 45, 47, 49–53, 55–60]
Emergency care visits	–		3.5	3.6	7.2	[67]	€153.07	[40–43, 45, 47–51, 51–56, 60, 63–65, 102, 103]
Tests								
Electrocardiogram	2.5	[10] and experts	2.5	3.5	4.0	[10]	€20.90	[42, 43, 46, 47, 49, 51, 53, 55, 57, 60–62]
Cardiopulmonary exercise test	1.0		1.0	1.0	1.0	[10]	€311.00	[42–46, 49–55, 57, 63]
Six-Minute Walk Test	1.0		2.5	3.5	4.0	[10]	€106.53	[46, 49, 51–54, 57, 64]
Echocardiogram	1.0		1.0	1.0	2.0	[10]	€103.87	[41–43, 45–47, 50, 52–57, 60, 61]
Biochemistry	2.5		2.5	3.5	4.0	[10]	€83.55	[43, 52, 53, 55, 58, 61]
Extended biochemistry	–		1.0	1.0	1.0	[10]	€188.17	[43, 46, 52–55, 57, 58, 61, 64]
Blood gas analysis/(V/Q Scan)	1.0/(1.0)		1.0	1.0	2.0	[10]	€44.50 (€331.04)	[42, 43, 45, 46, 54, 56, 57, 61]
Right heart catheterisation	1.0		1.0	1.0	1.0	[10]	€1,055.38	[41, 43, 46, 47, 51, 54, 60]
CT angiogram	1.0		1.0	1.0	1.0	Experts	€217.53	[42, 49, 52–57, 60, 104]
Oxygen saturation	–		2.5	3.5	6.0	[68]	€24.75	[46]
Chest X-ray	2.5		1.0	1.0	2.0	[68]	€20.00	[42, 43, 45–50, 52–57]
Hospitalisations	–		9.4	10.8	22.8	[67]	€601.22	[42, 43, 46, 47, 49–57, 60, 63]
Transplants	–		–	7.5	8.5	[69, 70]	€85,629.57	[79]

(1) The time for which the resources are estimated is annual. (2) Unit costs refer to the median costs of the first and follow-up visits at the different Spanish AACC. (3) The visit to the emergency department/unscheduled urgent visit to the referral centre is a weighting (40% and 60%, respectively). (4) The cost of lung scan is an average of the median unit costs of perfusion and ventilation lung scan. (5) For days of hospitalisation in FC I and II, a weighting of 10% and 90%, respectively, was assumed. (6) We assumed that a total of 16 PAH-lung transplants were performed during 2020 in Spain

Inpatient and outpatient visits to general and specialised practitioners were included, as well as nursing and emergency visits [10, 66–68]. For emergency visits, we assumed that 40% were to general emergency services, whilst 60% were unscheduled visits to the referral centre.

The tests required at diagnosis and follow-up were based on the guidelines for the diagnosis and treatment of pulmonary hypertension published by the European Society of Cardiology (ESC) and the European Respiratory Society (ERS) [10]. These included electrocardiograms, echocardiograms, biochemistry tests, cardiopulmonary exercise tests, the Six-Minute Walk Test, blood gas analyses, catheterisation, angiograms, and chest X-rays, among others. The experts specified the mean annual consumption of four types of tests per incident and prevalent patient.

The mean number of hospitalisation days, by FC, was based on information obtained in the literature [67]. As data on FC I-II was aggregated in the original source, we assumed a distribution of 10% of patients in FC I and 90% in FC II, based on the experts’ opinions. Data on resource use related to transplants were obtained from the 2020 report on lung transplants from the *Organización Nacional de Transplantes* (National Transplant Organisation, ONT), which included transplants performed on patients with pulmonary hypertension [69]. Based on literature data [70], we assumed that 47% of transplants were performed in FC III and the remaining, in FC IV.

For the pharmacological treatment of PAH, primary data based on routine clinical practice were used (Table 2). Doses were calculated according to routine clinical practice and product labels of each drug [71]. Administration costs, if applicable, were also included. The resources necessary for the administration of intravenous and subcutaneous drugs (hospital stays and training by the nursing staff) were approximated through information collected in the literature [72].

**Table 2:** Drugs per patient consumption and unit costs, by FC. Sources: Botplus (2021) [80], AEMPS (2021) [71], Roman (2012) [72] and others [42, 43, 46, 47, 49–57, 60, 63, 105]

Drug	Admins. route	Posology	Unit consumption	Pack cost	Presentation	Unit cost*	Total cost excl. adm. (patient/year)	Admin.cost (patient/year)	Total cost (patient/year)	Distribution of drug’s use			
										FC I-II (%)	FC III (%)	FC IV (%)	Total (%)
Sildenafil	O	20 mg, 3×/day	60 mg/day	€306.81	90 tabl	€0.17	€3,732.86	€0	€3,732.86	9.6	8.2	11.1	9.1
Tadalafil	O	40 mg, 1×/day	40 mg/day	€324.48	56 tabl	€0.29	€4,229.83	€0	€4,229.83	29.4	30.0	22.2	29.6
Riociguat	O	1–2,5 mg, 3×/day	3 tablets/day	€1,257.98	42 tabl	€29.95	€32,797.44	€0	€32,797.44	0.0	2.7	0.0	1.2
Bosentan	O	125 mg, 2×/day	250 mg/day	€124.80	56 tabl	€0.02	€1,626.86	€0	€1,626.86	14.7	6.4	0.0	10.7
Ambrisentan	O	5-10 mg, 1×/day	1 tablet/day	€859.04	30 tabl	€28.63	€10,451.65	€0	€10,451.65	15.4	9.1	11.1	12.6
Macitentan	O	10 mg, 1×/day	10 mg/day	€2,446.08	30 tabl	€8.15	€29,760.64	€0	€29,760.64	21.3	26.4	22.2	23.7
Selexipag (prevalents)	O	200–1,600 mcg, 2×/day	2 tablets/day	€3,717.17	60 tabl	€61.95	€45,225.54	€0 €	€45,225.54	2.9	8.2	0.0	5.1
Selexipag (incidents)			2,6 tablets/day				€59,102.97		€59,102.97				
Iloprost	Inh	5 mcg, 6–9×/day	37,5 mcg/day	€414.69	30 blisters	€1.38	€18,920.38	€65.00	€18,985.38	2.2	0.9	0.0	1.6
Treprostinil	IV	(1,5 vials 5 mg/ml) 1×/month	7,5 mg/ml/month	€5,094.34	1 vial	€1,018.87	€91,698.05	€195.00	€91,893.05	2.2	0.9	0.0	1.2
Epoprostenol	IV	0,5 mg (9 vials 0,5 mg) 1×/48 h	4,5 mg/48 h	€62.99	1 vial	€125.99	€103,465.67	€4,403.54	€107,869.21	2.2	7.3	33.3	5.1

(1) * EUR per mg. or EUR per tablet. (2) For selexipag, maintenance doses were assumed for prevalent patients. For incident patients, a titration period of 8 weeks was assumed, until reaching the maximum dose of 1,600 µg, 2 times a day. (3) Laboratory sale prices were considered, with official deductions and VAT. (4) O: oral. IV: intravenous route. Inh.: inhalation route. ×/day: times per day

#### Direct non-healthcare costs

DNHC included supportive therapies, medical devices, and formal and informal care. The consumption frequency of each of these items among patients with PAH was obtained from the HPE-ORG survey [39] (Table 3).

**Table 3:** DNHC resource consumption per patient during 1-year follow-up by FC, and unit costs

	Resource consumption (hours or % of patients in each FC who need this item)				Unit cost (€)	
	FC I-II	FC III	FC IV	Ref	EUR	Ref
Supportive therapies						
Rehabilitation/physiotherapy	8.1	2.0	3.2	[39]	€20.86	[40–43, 45, 47–55, 60]
Nutrition	0.6	0.0	0.8		€50.89	[42, 45, 49]
Psychology	1.6	4.0	2.4		€59.40	[45, 47, 49, 54, 57]
Social work	0.6	1.0	2.4		€46.97	[43, 52, 53, 63]
Oxygen therapy	22%	58%	67%		€7.00	[42, 45, 63, 104]
NIMV	0%	0%	13%		€247.41	[46]
Medical devices						
Wheelchair	3%	8%	13%	[39]	€258.15	[106–118]
Walker	3%	0%	0%		€75.53	
Adjustable bed	8%	17%	27%		€1,835.71	[119]
Personal care						
Informal care	277	581	675		€14.68	[81, 82]
Formal care	99	209	242		€16.25	

(1) Following Hawn (2020) [74], we have assumed a consumption of 365 days of oxygen therapy for those patients who need it, and an average of 12 days per year of hospitalisation due to the need to use Non Invasive Mechanical ventilation (NIMV). (2) Mixed care has been divided equally (50%/50%) between formal and informal care. (3) The average number of annual hours of formal and informal care were calculated by applying the percentage obtained from the HPE-ORG survey (26% and 74%, respectively)

Patients with PAH may require a comprehensive care approach with additional support from other healthcare professionals to improve activities of the daily living [73]. Accordingly, the mean annual consumption of supervised rehabilitation/physiotherapy, nutrition, psychotherapy, and social work sessions, as well as long-term oxygen therapy and non-invasive mechanical ventilation, were also included. This study assumed that patients used oxygen therapy on a daily basis (365 days per year) and that mechanical ventilation required 12 days of hospital stay [74]. In addition, the proportion of patients in need of a wheelchair, a walker, or an adjustable bed were also contemplated [39].

Regarding personal care, the percentage of patients within each FC who required formal, informal, or mixed care were extracted [39]. Personal care was divided equally into formal and informal care whenever the type of assistance was left unspecified. Moreover, based on assumptions of free time of each caregiver, the distribution of the caregivers’ time was defined [75].

#### Indirect costs

IC refer to the production lost to cessation or reduced productivity of the patient as a consequence of the morbidity and degree of disability. A human capital approach, which takes the patient perspective and counts any hour not worked as an hour lost [76, 77] was adopted to estimate productivity losses, including the reduction of working hours, working days lost to the disease, temporary and permanent leaves, and early retirement. Additionally, work time lost associated with the management of the disease was also considered (Table 4) (see Additional file 1 for more details).

**Table 4:** IC resource consumption during 1-year follow-up by FC, and unit costs

	Frequency of lost work (number of hours or % of patients)				Unit cost (€)	
	FC I-II	FC III	FC IV	Ref	EUR	Ref
Labour productivity losses						
Reduction of working hours	7%	0%	0%	[39]	6.14€	[105]
Work days lost due to disability	180	0	0	[18]	15.23€	[83]
Temporary leave	20%	0%	0%	[39]		
Permanent leave	53%	86%	100%	[39]		
Early retirement	7%	14%	0%	[39]		
**Total Time lost**	**119.1**	**138.1**	**358.4**			
Time lost due to medical visits	17.5	25.7	43.6	[66, 67]	15.23€	[83]
Time lost due to hospitalisations	74.9	87.4	183.4	[67]		
Time lost due to supportive services	17.7	13.0	113.4	[39]		
Time lost due to tests	9.0	12.0	18.0	[10, 68]		

(1) For early retirement and permanent disability leave, losses of 100% of working hours (1,581 h per year) were considered. (2) For temporary leave, 75% of the annual working hours (1,191 h) were considered. (3) In the HPE-ORG survey, out of 2 patients who reported a reduction in working hours, only 1 gave details on the size of this reduction (40%, 632 h per year). (4) In the study by Joish (2014), 180 h lost (29.6 days) were reported per patient with PAH. It was assumed that all these hours are lost by patients in FC I-II, since all patients in the other classes have a 100% loss due to other causes. (5) The cost / hour for all types of job loss is based on the annual wages of all occupations, both sexes, from the INE. The exception is the unit cost of the reduction of the working day, which is based on the average wages of all the part-time occupations of the INE

Information on the loss of labour productivity by FC (early retirement, permanent disability, temporary leave or reduction of working hours) was extracted from the HPE-ORG survey [39], as the studies identified by our literature review were not undertaken in Spain, nor displayed results disaggregated by functional class, nor provided enough detail to allow for the estimation of productivity losses [16–18, 24]. Work days lost to disability were only applied to patients in FC I-II [18], since, according to the HPE-ORG survey, all patients in the latter FC under 65 years of age who were working when they were diagnosed, had to leave their job (details on the methodological aspects on labour productivity can be found at Additional file 1). Despite not having enough details by FC provided in the literature, the results found in the HPE-ORG survey (100% of patients in FC III-IV are either retired or have asked for a permanent leave) seems to be in line with the studies regarding productivity losses in PAH patients, as they indicate this disease has a high impact over their working condition[16–18, 24].

The time spent on medical visits and tests, including the corresponding traveling time, was also considered. An average duration of 30 to 60 min was assumed for each visit/test, with an average travelling time of 1 h (round trip). For hospitalisations and NIMV, the average duration considered was 8 h per day (equivalent to one working day).

### Costs

Estimated costs were reported in 2020 Euros (€). Unit healthcare costs used were the median values of the latest published for each Autonomous Community in Spain. If specified on the respective Official Regional Bulletins, unit costs were updated to €2020 using the Consumer Price Index (CPI) [78]. The unit costs for non-pharmaceutical DHC are presented in Table 1.

The cost per hospitalisation day was calculated as the median cost of a medical or surgical hospitalisation day among the different Autonomous Communities [42, 43, 46, 47, 49–57, 60, 63]. The cost per transplant was the weighted average of that of a heart and lung transplant performed by the NHS [79].

Annual drug costs were calculated using mean dosages and list prices, including Royal Decree Law 8/2010 deduction rates, when necessary, a 4% of value-added tax (VAT) entitled for Spain [80], and administration costs, where applicable [81, 82] (Table 2).

The cost per supportive therapy service was calculated as the median cost per session among the different Autonomous Communities. Moreover, the median unit cost of a wheelchair and a walker was also collected from the regional official regulations, and the unit cost of the adjustable bed was approximated from the information contained in the public sector contracting platform (tenders), using the CPV code 33192100 (Table 3).

The unit cost of formal care was calculated as the average annual female salary of the health and social services subsector. For informal care, the average annual female salary of all sectors, was used. Average working hours were also obtained from INE [82].

Labour productivity losses were monetised based on annual wages and working hours for all occupations and both genders. The costs associated with these lost hours were calculated as the average annual salary per hour among all occupations in 2020, for both sexes [83] (details on the methodological aspects on labour productivity can be found at Additional file 1).

### Statistical analysis

Descriptive statistics were generated, reporting means and standard deviations for continuous variables, and counts and percentages for categorical variables. The average annual cost per patient was estimated, differentiating by FC, based on the annual consumption of each resource and its unit cost.

Separate estimations were performed for prevalent and incident patients. For prevalent patients, follow-up medical visits and tests were considered, and no drug administration cost was assumed [72]. For incident patients, according to current guidelines, diagnosis tests and first medical visits were assumed, regardless of the patient’s FC [10]. To this, the cost of care management and supportive therapies were added, assuming that incident patients consumed half the annual resources of prevalent ones, as the exact date of diagnosis couldn’t be specified (this was done in order to avoid applying an overestimated cost). Transplant costs were not included for incident patients, given the time it takes for patients to be included in the transplant program, which is followed by an extended time they spend on the waiting list [10]. Likewise, costs of early retirement, permanent disability, temporary leave, or reduction of working hours were not included, as applying for any of those procedures is highly bureaucratic, leading to long lead times until they are granted to patients [84]. The two types of work productivity loss considered for incident patients were work days lost and time spent on visits and transportation.

Finally, the total annual cost burden for the NHS and for society as a whole was calculated, multiplying the estimated average annual cost per patient by the number of prevalent and incident patients in 2020 in the country. A low and high prevalence range was applied (16.0 and 25.9, respectively [14, 85]) and an incidence of 3.7 [14] to the adult Spanish population of 39.1 million in 2020 [86]. Distinctions were also made by FC. All analyses were performed using Excel.

### Sensitivity analysis

A univariate deterministic sensitivity analysis was conducted to examine the uncertainty of the model. Twelve different scenarios were built and validated by the experts committee, based on the possible variations of key sensitive parameters and assumptions: (i) maximum prevalence (from 25.9 to 55 ppm) [87]; (ii) number of visits for diagnostic (± 40–50%); (iii) selected medical tests for diagnostic (± 60%); (iv) distribution of emergency visits (emergency services and unscheduled visits to referral from 0 to 100%); (v) distribution of number of hospitalization days by FC I-II (from 0 to 100%); (vi) discount on drugs (from 0 to 43.1%) [88]; (vii) unit costs for tests, visits, transplant, transport, hospitalisation and support therapies (minimum and maximum from the Autonomous Communities); (viii) number of hours dedicated by caregivers to patients, per week (± 28–33%); (ix) distribution of formal and informal care (formal: from 17 to 36%); (x) time spent on medical visits and tests (± 25–50%); (xi) wages and average working hours (± 5%); (xii) loss of productivity related to early retirement, permanent leave and temporary leave (± 10–20%) (see Additional file 2 for more details).

## Results

### Average cost per patient

The average total annual cost per prevalent patient with PAH was estimated at €98,839 (Table 5). DHC represented 62.5% (€61,739), followed by IC (25.7%; €25,369) and DNHC (11.9%; €11,731). Pharmacological treatment was the main cost item, responsible for 49.9% of the average total cost (ranging between 40.8% and 62.7% according to FC). Data showed that 32.1% of patients were on monotherapy, 45.5% on double therapy, and 22.4% on triple therapy. All patients in FC IV were on triple therapy, 11.1% among patients in FC I-II, and 63.0% among patients in FC III. Throughout one year, each patient consumed an average of 1.9 drugs, the two main drugs being tadalafil (29.6% of the total drugs used) and macitentan (23.7%), followed by ambrisentan (12.6%), bosentan (10.7%), sildenafil (9.1%), epoprostenol and selexipag (5.1% each), iloprost (1.6%), and riociguat and treprostinil (1.2% each). Following pharmacological treatment, loss of productivity due to permanent disability (18.6%; 11.5–19.9%), informal care (7.3%; 4.7–8.2%), and hospitalisations (6.8%; 6.3–8.5%) were also key cost items.

**Table 5:** Estimated annual average cost per patient with PAH in Spain by FC. Incident and prevalent patients

	Incident patient				Prevalent patient			
	FC I-II	FC III	FC IV	Total	FC I-II	FC III	FC IV	Total
Diagnosis	€2,882	€2,882	€2,882	€2,882	− €	− €	− €	− €
Medical visits	€539	€677	€1,179	€668	€1,078	€1,354	€2,358	€1,336
Tests	€377	€485	€628	€460	€2,530	€2,765	€3,101	€2,714
Hospitalisations	€2,778	€3,247	€6,854	€3,346	€5,555	€6,493	€13,708	€6,693
Transplants	− €	− €	− €	− €	− €	€1,274	€12,935	€1,673
Drugs	€13,825	€27,920	€69,836	€26,330	€26,619	€51,923	€130,866	€49,324
**DIRECT HEALTHCARE COSTS**	**€20,401**	**€35,210**	**€81,378**	**€33,686**	**€35,783**	**€63,810**	**€162,967**	**€61,739**
Formal care	€807	€1,695	€1,969	€1,432	€1,614	€3,390	€3,937	€2,863
Informal care	€2,031	€4,268	€4,956	€3,604	€4,063	€8,535	€9,912	€7,208
Supportive therapies	€605	€1,071	€1,414	€947	€881	€1,817	€2,465	€1,564
Medical devices	− €	− €	− €	− €	€53	€109	€175	€96
**DIRECT NON-HEALTHCARE COSTS**	**€3,444**	**€7,034**	**€8,338**	**€5,982**	**€6,558**	**€13,742**	**€16,314**	**€11,731**
Early retirement	− €	− €	− €	− €	€1,605	€3,440	− €	€2,619
Permanent leave	− €	− €	− €	− €	€12,843	€20,641	€24,081	€18,399
Temporary leave	− €	− €	− €	− €	€3,629	− €	− €	€1,154
Working hours reduction	− €	− €	− €	− €	€259	− €	− €	€82
Working days missed	€914	€1,371	€2,742	€1,320	€2,742	− €	− €	€872
Work time lost due to visits/test and trips	€907	€1,051	€2,729	€1,121	€1,815	€2,103	€5,458	€2,243
**INDIRECT COSTS**	**€1,821**	**€2,422**	**€5,471**	**€2,441**	**€22,892**	**€26,184**	**€29,539**	**€25,369**
**TOTAL COSTS**	**€25,666**	**€44,667**	**€95,188**	**€42,110**	**€65,233**	**€103,736**	**€208,821**	**€98,839**

The average annual cost per incident patient with PAH was estimated at €42,110. DHC represented 80.0% (€33,686). Drugs were also the main type of cost, being responsible for 62.5% of the average total cost (ranging between 53.9% and 73.4% according to FC), followed by the costs of informal care (8.6%; 5.2–9.6%) and hospitalisations (7.9%; 7.2–10.8%).

Higher FCs were associated with increased costs. The average cost per prevalent patient in FC IV (€208,821) was 2.0 and 3.2 times higher, than costs in FC III (€103,736) and FC I-II (€65,233), respectively. For incident patients, the average costs in FC IV (€95,188) was 1.7 and 3.7 times higher than the costs in FC III (€44,667) and FC I-II (€25,666).

The increase in costs by FC was observed in all cost categories. DHC for prevalent patients were €35,783 in FC I-II, €63,810 FC III and €162,967 in FC IV, whilst for incident patients, those costs were €20,401; €35,210 and €81,378, respectively. DNHC costs were €6,558 for prevalent patients in FC I-II, €13,742 in FC III and €16,314 for FC IV. For incident patients, those costs were €3,444 (FC I-II), €7,034 (FC III) and €8,338 (FC IV). IC costs were €22,892; €26,184 and €29,539 for prevalent patients in FC I-II, FC III and FC IV, respectively, and for incident patients, those costs were €1,821; €2,422 and €5,471.

### Total costs

The distribution of cases by FC was extracted from the proportions observed in the REVEAL and Spanish national registries, that is 32% of patients in FC I-II, 61% in FC III, and 7% in FC IV [89, 90].

Based on epidemiological data and the estimations of average costs per patient, the economic burden of PAH in Spain was estimated to be between €67,891,405 and €106,131,626 in 2020 (Table 6). Prevalent patients represented 91–94% of these costs (between €61,802,379 and €100,042,600). DHC were estimated to be between €43,475,515 and €67,362,079 (64% of the total cost, for both low and high value estimates), whereas IC were estimated to be between €16,215,758 and €26,030,824 per year (24%) and DNHC ranged between €8,200,132 and €12,738,724 (12%).

**Table 6:** Estimated total costs of patients with PAH in Spain by FC. Lower-end and higher-end of the patient prevalence range

	Lower-end of prevalence range (16.0 per adult pop.)				Higher-end of prevalence range (25.9 per adult pop.)			
	FC I-II	FC III	FC IV	Total	FC I-II	FC III	FC IV	Total
Diagnosis	€132,501	€255,418	€28,750	€416,669	€132,501	€255,418	€28,750	€416,669
Medical visits	€239,242	€578,916	€113,487	€931,644	€371,931	€899,995	€176,429	€1,448,355
Tests	€520,369	€1,102,978	€140,064	€1,763,411	€831,613	€1,758,862	€222,853	€2,813,328
Hospitalisations	€1,232,335	€2,776,603	€659,802	€4,668,739	€1,915,814	€4,316,567	€1,025,742	€7,258,124
Transplants	− €	€488,300	€558,057	€1,046,357	− €	€790,435	€903,354	€1,693,790
Drugs	€5,928,730	€22,377,032	€6,342,933	€34,648,694	€9,203,790	€34,691,530	€9,836,494	€53,731,814
**DIRECT HEALTHCARE COSTS**	**€8,053,177**	**€27,579,246**	**€7,843,092**	**€43,475,515**	**€12,455,649**	**€42,712,807**	**€12,193,623**	**€67,362,079**
Formal care	€358,021	€1,449,817	€189,515	€1,997,353	€556,588	€2,253,917	€294,624	€3,105,129
Informal care	€901,272	€3,649,725	€477,078	€5,028,076	€1,401,138	€5,673,942	€741,676	€7,816,756
Supportive therapies	€203,078	€791,318	€120,470	€1,114,867	€311,516	€1,222,181	€186,281	€1,719,978
Medical devices	€10,463	€41,839	€7,535	€59,837	€16,937	€67,727	€12,197	€96,861
**DIRECT NON-HEALTHCARE COSTS**	**€1,472,835**	**€5,932,700**	**€794,598**	**€8,200,132**	**€2,286,178**	**€9,217,767**	**€1,234,778**	**€12,738,724**
Early retirement	€319,221	€1,318,614	− €	€1,637,834	€516,738	€2,134,506	− €	€2,651,244
Permanent leave	€2,553,765	€7,911,681	€1,038,973	€11,504,419	€4,133,907	€12,807,034	€1,681,837	€18,622,778
Temporary leave	€721,526	− €	− €	€721,526	€1,167,970	− €	− €	€1,167,970
Working hours reduction	€51,451	− €	− €	€51,451	€83,286	− €	− €	€83,286
Working days missed	€587,151	€121,503	€27,353	€736,007	€924,451	€121,503	€27,353	€1,073,306
Work time lost due to visits/test and trips	€402,525	€899,277	€262,719	€1,564,521	€625,774	€1,398,035	€408,429	€2,432,238
**INDIRECT COSTS**	**€4,635,639**	**€10,251,074**	**€1,329,045**	**€16,215,758**	**€7,452,127**	**€16,461,077**	**€2,117,620**	**€26,030,824**
**TOTAL COSTS**	**€14,161,651**	**€43,763,019**	**€9,966,735**	**€67,891,405**	**€22,193,954**	**€68,391,651**	**€15,546,021**	**€106,131,626**

More than 80% of the burden was attributed to four types of costs: drug treatment (€34,648,694–€53,731,814; 51% of the total), loss of labour productivity due to permanent disability (€11,504,419–€18,622,778; 17%), informal care (€5,028,076–€7,816,756; 7%), and hospitalisations (€4,668,739–€7,258,124; 7%).

The total costs associated with patients in FC I-II ranged between €14,161,651 and €22,193,954, while patients in FC III represented between €43,763,019 and €68,391,651, and patients in FC IV between €9,966,735 and €15,546,021.

### Sensitivity analysis

**Fig. 1:**
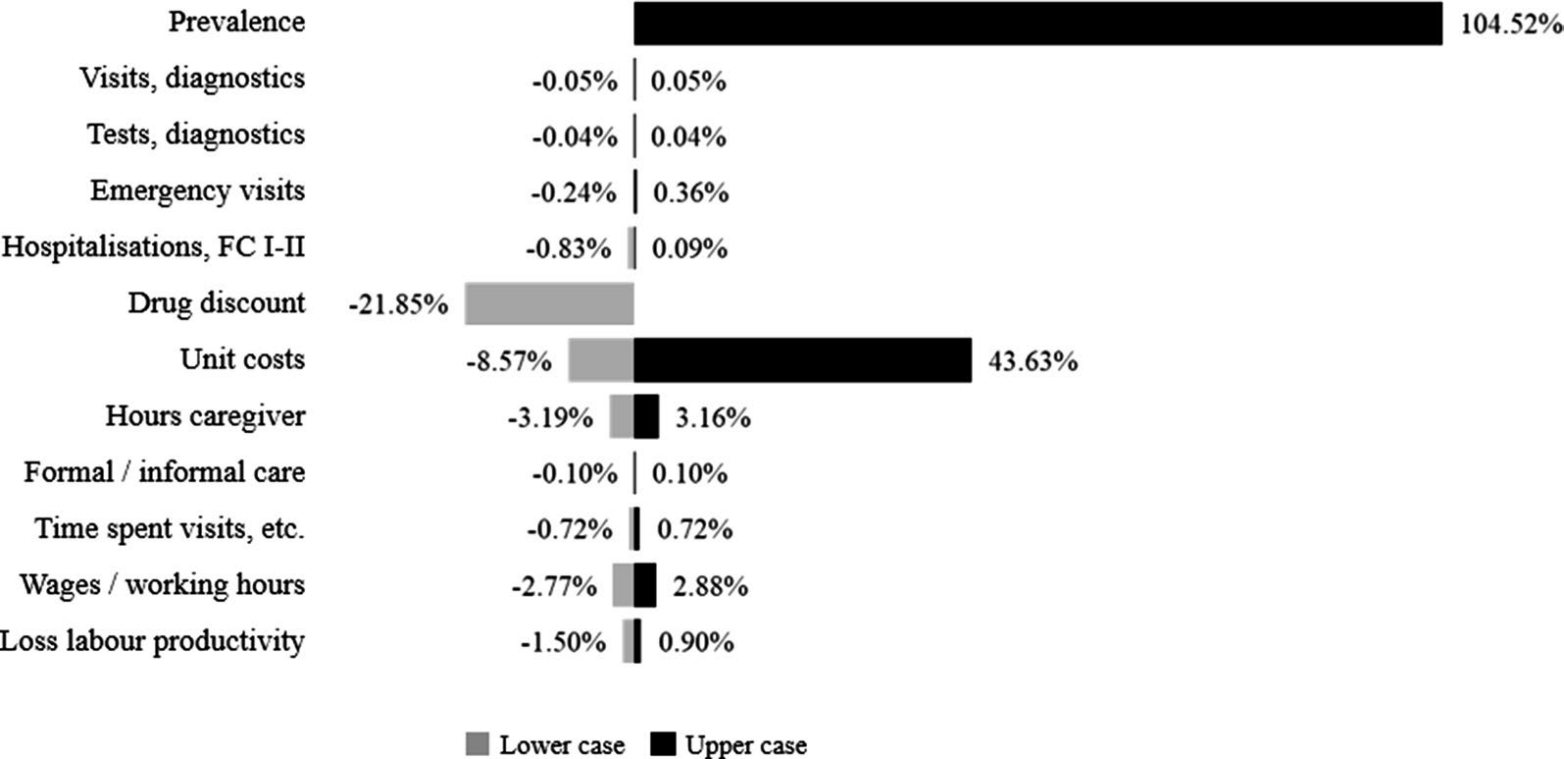
Tornado diagram, % of variation in total costs vs. base case

The sensitivity analysis showed that the most sensitive parameter was prevalence, with an impact of 105% (+ €111 million versus base case) over total costs estimated. Changes in unit costs could derive in − 9% (€9 million) or + 44% (€46 million). A drug discount could reduce total costs up to 22% (€23 million). The other parameters varied at a median of between -0.7% and + 0.4% (ranging from -3% to + 3%) (Fig. 1) (see Additional file 2 for more details).

## Discussion

In most publicly funded systems, resource allocation is a pressing issue provided that there is an opportunity cost associated with every resource spent on a disease or a person. Achieving more efficient and equitable health systems requires a deeper knowledge of the global burden of each disease and of the economic and social consequences of changes in its management. Traditional methods to assess the economic and social impact of a disease and its management may be especially useful in the field of rare diseases, where there is a lower degree of knowledge and greater heterogeneity and uncertainty around the evidence found in the literature [91]. In fact, a growing number of patient associations are carrying out studies to measure and increase the awareness of the economic and social impact of these diseases [92, 93]. Furthermore new methodologies, such as the multi-criteria decision analysis, are being progressively used by decision-makers worldwide to widen the set of criteria considered in evaluation processes and to allow the assessment of drugs from an holistic perspective [94–96].

To our knowledge, this is the first study to quantify the annual economic burden of PAH in Spain. A thorough analysis of the different types of costs borne by both patients and their environment, as well as the public system and society as a whole has allowed the comprehensive quantification of the total costs associated with this rare disease in Spain, which ranged between €67.9 million and €106.1 million per year. More specifically, DHC accounted for €43 million to €67 million (64% of the total cost), while IC accounted for €16 million to €26 million (24%) and DNHC for €8 million to €13 million (12%). In terms of the average cost per patient, a newly diagnosed patient had a total average cost of €42,110 per year, while a prevalent one was associated with an average cost of €98,839 per year.

Our results are in line with other PAH published studies. According to a systematic review carried out in 2016, which included eight studies that evaluated the economic burden of PAH in Germany and the United States [97], the median DHC of these patients were estimated at $65,370 per year, similar to the €61,739 of DHC estimated in this study for prevalent patients. Six of these studies were conducted from the payer's perspective [19, 27–30, 98], while two of them further included the patient's perspective, with co-payments, medical devices, and home modifications [20, 24]. None of the studies assessing the economic burden of PAH included IC.

Moreover, these studies reported annual DHC ranging between $29,700 and $143,000 per patient [19, 20, 24, 27–30, 98]. The large variability was due to differences in study design (prospective versus retrospective approaches), sources of information, cost items considered, time horizon, focus, and perspective of the analysis, in addition to country-related differences such as health systems and unit prices (e.g., Germany versus USA) [97]. Moreover, substantial differences in patient characteristics in terms of age, comorbidities, and FC, were further observed.

Furthermore, pharmacological costs were contemplated in all studies, though from different approaches and scopes. In Germany, Wilkens et al. (2010) analysed the costs associated with 118 patients, of which 64% received monotherapy, 19% double therapy, 5% triple therapy, and 2.5% intravenous treatment [24]. They concluded that drugs were the most important cost item, accounting for 96% of total direct costs on average. Other studies obtained similar results, especially regarding combination therapies [28, 98]. Nevertheless, some studies found that after applying a certain treatment, new drugs could have an economic benefit or an offset effect on other DHC, by reducing medical visits and hospitalisations [27, 29]. Other studies recalled that both an improved understanding of PAH pathogenesis and specific therapies have helped to decrease mortality over the past 20 years [11].

It should be noted that the published evidence further confirms that costs increase with the severity of the disease, and that closer monitoring of the patient can reduce costs [24, 98]. However, few studies have analysed the differences in costs per FC. One of these studies estimated a greater cost for patients in FC IV compared to those in FC II (+ 27%) [24]. In contrast, the present study estimated that the average DHC of a prevalent patient in FC IV is 4.6 times higher than that of a prevalent patient in FC I-II (or 3.5 times higher excluding pharmacological costs).

Finally, the evidence also highlights the high economic burden posed by PAH in terms of work impact, with 32% of patients having to retire from work due to the disease [24]. Furthermore, at the time of diagnosis, between 40 to 50% of patients with PAH were already not working and, after diagnosis, 74–84% of those employed had their working conditions affected somehow, through reduced working hours, missed days of work, need for retirement, long term absence leave or simply because those patients decided to give up work completely[16–18, 24]. Our study estimated that 90.7% of the patients had their working life affected, which is in line with the results from the literature.

One of the main strengths of this study is the broad perspective applied to the analysis. In fact, this is also the first study that has attempted to approximate the IC derived from this rare disease. DHC accounted for the largest part of the total cost, 64%, but the study has shown that IC (24%) and DNHC (12%) account for considerable amounts, ranging from €24.4 million to €38.8 million per year, depending on the prevalence considered. Performing this study from a social perspective allowed for a broader and more realistic quantification of what the disease really means in the country, not only for the health system but for all the agents involved. In addition, the high degree of detail of the cost items included in our analysis allows for a better visualisation of the problems that these patients face in their day-to-day life, with the disease having a considerable impact on the physical, emotional, and working domains. According to a scoping review of cost-of-illness studies conducted on rare diseases, costs due to productivity losses, non-medical costs, and informal care costs were quantified in 68%, 60%, and 43% of studies, respectively [91].

Moreover, stratifying the economic and social impact of PAH according to FC confirmed that increased disease severity was associated with an increase in all cost items, including IC. According to the present study, the average cost of a prevalent patient in FC IV is twice that of a patient in FC III (€208,821 versus €103,736) and more than three times that of a patient in FC I-II (€65,233). In global terms, patients with the worst functional status accounted for only 6.9% of the adults suffering from PAH in Spain, but they were responsible for 14.7% of total costs, while patients with FC I-II, which represent a third of the total were responsible for 20.9% of the costs. Another element of interest, since it is not usually included in studies of economic burden, is the distinction between incident and prevalent patients.

To contextualize the results, it would be convenient to analyze other economic burden studies carried out in Spain. There is a reduced number of recent published studies comparable to ours in terms of information collected. According to their estimations, the annual average cost per patient ranged between €1,100 (low back pain) and €478,000 (transthyretin amyloid polyneuropathy) [38, 99]. In terms of total burden, the total annual cost associated with PAH could be much lower than that of pathologies such as multiple sclerosis, depression or low back pain [34, 99, 100].

However, the present study is not exempt from certain limitations. The first refers to the uncertainty associated with the information used as the basis for the analysis. In this regard, data on resource consumption were not collected retrospectively, but from the literature. Accordingly, some assumptions in agreement with the expert committee were used. On the other hand, the primary data used to estimate the costs of pharmacological treatment, DNHC and IC may not be fully representative of the group of patients with PAH in Spain. Additionally, official drug prices do not include potential discounts applied after negotiations between suppliers and hospitals. Moreover, the costs derived from having a poorer quality of life or the intangible costs associated with the disease (e.g., pain, anxiety, etc.) were not included in the analysis. Accordingly, our estimations reflect only a part of the real social impact of the disease. Finally, the prevalence of PAH in Spain could be underestimated, as other more recent European sources stablish a higher prevalence. We tried to manage this challenge by also using an international reference to capture the potentially higher real prevalence. Considering that the maximum prevalence reported in Europe amounts to 55 cases per million habitants [87], if real prevalence in Spain was closer to this numbers, total annual cost would increase up to €217 million. This potential variability suggests that an integrated updated national PAH registry would be necessary.

## Conclusions

Patients with PAH represent a substantial financial burden for the health system and society at large, derived not only from their clinical management, but also from the need of personal care and/or supportive therapy, and the labour impact caused by the disease. Non-pharmacological costs have a considerable weight in PAH total costs, with the social dimension accounting for up to one third of the total economic impact, so limiting the perspective of decisions to direct healthcare costs can lead to significant biases.

Our results reinforce that the therapeutic objective of preventing the disease from progressing and, whenever possible, following a multidisciplinary approach that allows a closer monitoring of patients and their maintenance in less severe FC levels, should be pursued, as it would translate into clinical, but also economic and social benefits. In order to better plan for resource allocation, it would also be convenient to consider the costs throughout the patient's lifetime, and not just in a given year, and to propose strategies that allow long-term savings.

Finally, investing on optimising databases is necessary to have up-to-date, reliable, and unified information sources that allow to determine more accurately the impact of the disease. A coordinated effort should be made to enhance the fulfilment and update of PAH registries on a national level and to investigate the real impact of the disease on the patient’s daily life. Future research could be conducted to broaden the scope of this analysis and help overcome the methodological difficulties of the present study.

## Supplementary Information

**Additional file 1.** Methodological aspects of labour productivity.

**Additional file 2.** Sensitivity analysis.

## Data Availability

The datasets generated and/or analysed during the current study are not publicly available due to confidentiality issues and to safeguard accurate data interpretation, although they are available from the corresponding author on reasonable request.

## Abbreviations

AACC: Autonomous Communities
CPI: Consumer Price Index
CPV: Common Procurement Vocabulary
DHC: Direct Healthcare Costs
DNHC: Direct Non-Healthcare Costs
ESC: European Society of Cardiology
ERS: European Respiratory Society
FC: Functional Class
HPE-ORG: Patient Organisation for Pulmonary Hypertension in Spain
IC: Indirect Costs
INE: National Institute of Statistics
NHS: National Health System
ONT: National Transplant Organisation
NIMV: Non-Invasive Mechanical ventilation
PAH: Pulmonary Arterial Hypertension
VAT: Value-Added Tax

## Declarations

### Published abstract

An Abstract of this work has been published at ISPOR Europe 2021 [101].
