# Supplementary material for: The economic burden of pulmonary arterial hypertension in Spain
Source: BMC Pulm Med. 2022 Mar 26;22:105. doi: 10.1186/s12890-022-01906-2 (PMC8962538; doi:10.1186/s12890-022-01906-2)
Supplement: Supplementary file 1 — Additional file 1. Methodological aspects of labour productivity. [file 12890_2022_1906_MOESM1_ESM.docx]

**THE ECONOMIC BURDEN OF PULMONARY ARTERIAL HYPERTENSION IN SPAIN**

**Authors**: Néboa Zozaya^1,2^, Fernando Abdalla^1^, Ignacio Casado Moreno^3^, Carlos Crespo-Diz^4^, Ana M. Ramírez Gallardo^5^, Joaquín Rueda Soriano^6^, Macarena Alcalá Galán^7^, Álvaro Hidalgo-Vega^8,9^

**Affiliations**: ^1^Department of Health Economics, Weber. Madrid, Spain; ^2^Department of Quantitative Methods in Economics and Management. University Las Palmas de Gran Canaria. Las Palmas, Spain; ^3^Pneumology Unit, University Hospital Virgen de las Nieves. Granada, Spain; ^4^Pharmacy Department. Complexo Hospitalario Universitario de Pontevedra. Instituto de Investigación Sanitaria Galicia Sur (IISGS), Pontevedra, Spain; ^5^Pulmonary Hypertension Unit, Hospital Clínic. Barcelona, Spain; ^6^Department of Cardiology, Hospital Universitari i Politècnic La Fe. Instituto de Investigación Sanitaria La Fe. CIBERCV, Valencia, Spain; ^7^Market Access Department, Janssen, Madrid, Spain; ^8^Weber Foundation. Madrid, Spain; ^9^Department of Economic Analysis and Finances. University of Castilla-La Mancha. Toledo, Spain

**SUPPLEMENTARY FILE 1: METHODOLOGICAL ASPECTS ON LABOUR PRODUCTIVITY**

**Calculation of labour productivity losses: prevalent patients**

For the calculation of labour productivity losses, we have followed the human capital approach. This method, which is the most used in cost-of-illness studies, considers the patient´s hours of productivity that are lost and calculates productivity costs as the product of total hours lost with hourly wage. Under this method, every hour not worked is an hour lost [1]. Below we describe the rationale and steps taken for the calculation of labour productivity losses.

Prior to that, it is important to highlight that studies yielded in the literature review including loss of labour productivity were not from Spain, nor showed disaggregated data per functional class, nor provided enough detail to allow for the estimation of productivity losses [2–5]. Some of the conclusions that were drawn from the literature review were: (i) at the time of diagnosis, 40%-50% of the patients are already not working (ii) after diagnosis, or at the time point in which the studies were conducted, out of those employed, 74%-84% of patients had their working conditions affected somehow (reduced hours, missed work, had to retire, asked for long term absence or decided to give up work completely).

Given that not enough details were found on the literature, we have used data from the survey which was carried out by *Hipertensión Pulmonar España Organización de Pacientes* (Spanish Pulmonary Hypertension Patient Organisation, HPE-ORG) [6] for the estimation of labour productivity loss in our study (for all functional classes). Calculations were done according to the following methodology:

1. In total, 64 Spanish patients with PAH answered the survey. Out of those, 20 were excluded for the labour productivity analysis as: (i) patient was not working when diagnosed, or (ii) patient did not answer the question related to productivity losses, or (iii) patient was older than 65, hence not in a working age.
2. Out of the 44 patients included in the labour productivity analysis, 30 (68%) were in FC I-II, 7 (16%) in FC III and 7 (16%) in FC IV.
   1. The 30 patients in FC I-II had the following working situation: 2 (7%) asked for reduction in working hours, 6 (20%) asked for a temporary leave, 16 (53%) asked for a permanent leave due to the disease and 2 (7%) asked for an early retirement. 4 patients (13%) did not have their working conditions affected.
   2. The 7 patients in FC III had the following working situation: 6 (86%) reported they had to request a permanent leave due to the disease and 1 (14%) retired.
   3. The 7 patients in FC IV had the following working situation: 7 (100%) reported they had to request a permanent leave due to the disease.
3. The % of ii (a, b and c) are stated in table 4 in the manuscript, under the heading “labour productivity losses”.
4. For permanent leave and early retirement, we considered losses of 100% of working hours in 1 year, which is 1,581 hours (stated in the notes from Table 4 in the manuscript), according to the number of hours worked by an employee in Spain (considering data from the National Statistics Institute, all occupations, both genders [7]).
5. For temporary leave, we considered losses of 75% of total working hours (1,581*75% = 1,191 hours, stated in the notes from Table 4 in the manuscript). We have used the 75% based on the actual reported temporary leave from the patients on the survey. Out of the 6 patients who asked for a temporary leave, 4 requested it for two years, 1 for 90 days and 1 for 100 days. The formula we used to calculate the 75% was: ((4*100%)+(1*90/365)+(1*100/365))/6
6. For the reduction of working hours, we considered losses of 40% of total working hours (1,581*40% = 632, stated in the notes from Table 4 in the manuscript). We have used the 40% based on the actual reported reduction of hours from the patients on the survey. Out of the 2 patients who had a reduction of working hours, only 1 stated the size of this reduction, stating they only work 3 days a week (do not work 2 days = 2/5=40%).
7. The price per hour lost applied was 15.23€, based on the National Statistics Institute, all occupations, both genders [8]. The only exception was in relation to reduction of working hours, which we applied 6.14€ as an hourly cost, based on the National Statistics Institute, partial time labour, both genders [9]. This is stated in Table 4 in the original manuscript.
8. The calculation of the cost per patient was done by multiplying the % of patients, times the number of hours, times the cost per hour.
9. Additionally, according to Joish (2014)[5] patients with PAH miss 29.6 days every year due to the disease. We have applied those 29.6 days to FC I-II as 100% of patients in FC III-IV are either on permanent leave or early retirement. To the 29.6 days, we applied the number of hours loss per day, according to the National Institute of Statistics (1,581/52/5 = 6,08 effective working hours), arriving to the 180 hours lost per year (29.6*6.08 = 179.97 [to which we applied a cost of 15.23€]).

In addition, as part of labour productivity losses, we have estimated the work time lost due to visits/tests and trips. The methodology used for this estimation was to multiply the number of visits and tests done per year by the sum of travel time plus the actual visit time. To the resulting total number of hours lost, we applied the cost of 15.23€ per hour, according to the National Statistic Institute.

The details are described in tables (S1).1-6 below. We follow the same order as per Table 4 in the manuscript.

**Table (S1).1: Reduction of working hours**

|  | **FC I-II** | **FC III** | **FC IV** |
| --- | --- | --- | --- |
| % of patients | 7% | 0% | 0% |
| Time lost / patient / year | 632 | n.a. | n.a. |
| Cost / hour lost | 6.14€ | n.a. | n.a. |
| Cost per patient per year | 259€ | 0€ | 0€ |

**Table (S1).2: Work days lost due to disability (working days missed)**

|  | **FC I-II** | **FC III** | **FC IV** |
| --- | --- | --- | --- |
| Time lost / patient / year | 180 | 0 | 0 |
| Cost / hour lost | 15.23€ | n.a. | n.a. |
| Cost per patient per year | 2,742€ | 0€ | 0€ |

**Table (S1).3: Temporary leave**

|  | **FC I-II** | **FC III** | **FC IV** |
| --- | --- | --- | --- |
| % of patients | 20% | 0% | 0% |
| Time lost / patient / year | 1,191 | n.a. | n.a. |
| Cost / hour lost | 15.23€ | n.a. | n.a. |
| Cost per patient per year | 3,629€ | 0€ | 0€ |

**Table (S1).4: Permanent leave**

|  | **FC I-II** | **FC III** | **FC IV** |
| --- | --- | --- | --- |
| % of patients | 53% | 86% | 100% |
| Time lost / patient / year | 1,581 | 1,581 | 1,581 |
| Cost / hour lost | 15.23€ | 15.23€ | 15.23€ |
| Cost per patient per year | 12,843€ | 20,641€ | 24,081€ |

**Table (S1).5: Early retirement**

|  | **FC I-II** | **FC III** | **FC IV** |
| --- | --- | --- | --- |
| % of patients | 7% | 14% | 0% |
| Time lost / patient / year | 1,581 | 1,581 | n.a. |
| Cost / hour lost | 15.23€ | 15.23€ | n.a. |
| Cost per patient per year | 1,605€ | 3,440€ | 0€ |

**Table (S1).6: Work time lost due to visits / tests / trips**

|  | **FC I-II** | **FC III** | **FC IV** |
| --- | --- | --- | --- |
| Time lost / patient / year | 119.1 | 138.1 | 358.4 |
| Cost / hour lost | 15.23€ | 15.23€ | 15.23€ |
| Cost per patient per year | 1,815€ | 2,103€ | 5.458€ |

**Calculation of labour productivity losses: Incident patients**

We have not considered costs related to reduction of working hours, temporary leave, permanent leave and early retirement for incidence patients. The rationale for not including them was: (i) incident patients were considered to be diagnosed in the middle of the year; (ii) the application for any of those changes in working conditions are followed by a period of administrative / bureaucratic steps which usually take a long time until final decision is made [10].

Hence, for incident patients, we have only considered costs for work days lost due to disability, and assumed that an incident patient in CF IV lost the same amount as a prevalent patient in CF I-II (2,742€per patient per year), whilst an incident patient in CF III lost half of the amount as a prevalent patient in CF I-II (1,371€per patient per year), and finally, an incident patient in CF I-II lost one third of the amount of a prevalent patient in CF I-II (914€ per patient per year).

In addition, for the work time lost due to visits/tests/trips for incident patients, we have considered half of the costs of the prevalent patients, as incident patients were assumed to enter in the middle of the year.

# **References**

1. van den Hout WB. The value of productivity: human-capital versus friction-cost method. Ann Rheum Dis. 2010;69:i89-91.

2. Armstrong I, Billings C, Kiely DG, Yorke J, Harries C, Clayton S, et al. The patient experience of pulmonary hypertension: a large cross-sectional study of UK patients. BMC Pulm Med. 2019;19:67.

3. Helgeson SA, Menon D, Helmi H, Vadlamudi C, Moss JE, Zeiger TK, et al. Psychosocial and Financial Burden of Therapy in USA Patients with Pulmonary Arterial Hypertension. Diseases. 2020;8.

4. Wilkens H, Grimminger F, Hoeper M, Stähler G, Ehlken B, Plesnila-Frank C, et al. Burden of pulmonary arterial hypertension in Germany. Respir Med. 2010;104:902–10.

5. Joish VN, Kreilick C, Germino R, Muccino D. Estimation Of Pulmonary Arterial Hypertension On Productivity Losses In The United States. Value Health. 2014;17:A118.

6. La carga socioeconómica de la hipertensión pulmonar en España. Hipertensión Pulmonar. https://hipertension-pulmonar.com/la-asociacion/estudios/255-la-carga-socioeconomica-de-la-hipertension-pulmonar-en-espana. Accessed 28 Jun 2021.

7. Instituto Nacional de Estadística (INE). Sexo y grupos principales de ocupación(28186). INE. https://www.ine.es/jaxiT3/Tabla.htm?t=28186&L=0. Accessed 26 Jan 2022.

8. Instituto Nacional de Estadística (INE). Ganancia media anual por trabajador. Sexo y grupos principales de ocupación(28186). INE. https://www.ine.es/jaxiT3/Tabla.htm?t=28186&L=0. Accessed 26 Jan 2022.

9. Instituto Nacional de Estadística (INE). Salarios medios por tipo de jornada, sexo y decil.(13927). INE. https://www.ine.es/jaxiT3/Tabla.htm?t=13927&L=0. Accessed 26 Jan 2022.

10. Ministerio de Inclusión, Seguridad Social y Migraciones. Seguridad Social: Trámites y Gestiones. https://www.seg-social.es/wps/portal/wss/internet/InformacionUtil/44539. Accessed 26 Jan 2022.
