# Supplementary material for: The economic burden of pulmonary arterial hypertension in Spain
Source: BMC Pulm Med. 2022 Mar 26;22:105. doi: 10.1186/s12890-022-01906-2 (PMC8962538; doi:10.1186/s12890-022-01906-2)
Supplement: Supplementary file 2 — Additional file 2. Sensitivity analysis. [file 12890_2022_1906_MOESM2_ESM.docx]

**THE ECONOMIC BURDEN OF PULMONARY ARTERIAL HYPERTENSION IN SPAIN**

**Authors**: Néboa Zozaya^1,2^, Fernando Abdalla^1^, Ignacio Casado Moreno^3^, Carlos Crespo-Diz^4^, Ana M. Ramírez Gallardo^5^, Joaquín Rueda Soriano^6^, Macarena Alcalá Galán^7^, Álvaro Hidalgo-Vega^8,9^

**Affiliations**: ^1^Department of Health Economics, Weber. Madrid, Spain; ^2^Department of Quantitative Methods in Economics and Management. University Las Palmas de Gran Canaria. Las Palmas, Spain; ^3^Pneumology Unit, University Hospital Virgen de las Nieves. Granada, Spain; ^4^Pharmacy Department. Complexo Hospitalario Universitario de Pontevedra. Instituto de Investigación Sanitaria Galicia Sur (IISGS), Pontevedra, Spain; ^5^Pulmonary Hypertension Unit, Hospital Clínic. Barcelona, Spain; ^6^Department of Cardiology, Hospital Universitari i Politècnic La Fe. Instituto de Investigación Sanitaria La Fe. CIBERCV, Valencia, Spain; ^7^Market Access Department, Janssen, Madrid, Spain; ^8^Weber Foundation. Madrid, Spain; ^9^Department of Economic Analysis and Finances. University of Castilla-La Mancha. Toledo, Spain

**SUPPLEMENTARY FILE 2: SENSITIVITY ANALYSIS**

**Rationale for the selection of parameters**

The results of this study were established according to different assumptions which were based on official sources, literature, clinical guidelines, surveys, routine clinical practice as well as experts’ opinion. In order to test the robustness of those assumptions, we have performed a univariate deterministic sensitivity analysis. The criterion for the selection of the parameters was to include all of those which were based on experts’ opinion (hence, lower level of evidence in comparison to the other sources) as well as other parameters which are considered as key variables. **Table (S2).1** describes all assumptions made, the source for each of them, and what parameters were included in the sensitivity analysis.

**Table (S2).1: Model assumptions, sources and parameters included in the sensitivity analysis**

| **Domain** | **Assumption** | **Sources** | **Rationale of included parameters** |
| --- | --- | --- | --- |
| Unit costs and working hours | Unit costs of the different resources analysed were estimated through rates and salaries in Spain: **(i)** unit healthcare costs used were the median values of the latest published for each Autonomous Community in Spain; (ii) if specified on the respective Official Regional Bulletins, unit costs were updated to €2020 using the Consumer Price Index (CPI); **(iii)** the cost per hospitalisation day was calculated as the median cost of a medical or surgical hospitalisation day among the different Autonomous Communities. **(iv)** the cost per transplant was the weighted average of that of a heart and lung transplant performed by the NHS; **(v)** annual drug costs were calculated using mean dosages and list prices, including Royal Decree Law 8/2010 deduction rates, when necessary, and a 4% of value-added tax (VAT) entitled for Spain; **(vi)** the median unit cost of a wheelchair and a walker was also collected from the regional official regulations, and the unit cost of the adjustable bed was approximated from the information contained in the public sector contracting platform (tenders), using the CPV code 33192100; **(vii)** the unit cost of formal care was calculated as the average annual female salary of the health and social services subsector. For informal care, the average annual female salary of all sectors, was used; **(viii)** average working hours were obtained from the National Statistical Institute | Different regional regulations or in official national sources [1–26], consumer price index [27], cost per transplant [28], value added tax for Spain [29], working hours from the National Statistics Institute [30], wages from the National Statistics Institute [31] | Included in the sensitivity analysis (key parameters): (a) Unit cost for tests, visits, transplant, transport, hospitalisation and support therapies; (b) wage and average working hours; (c) discount on drug costs. |
| DHC | For emergency visits, we assumed that 40% were to general emergency services, whilst 60% were unscheduled visits to the referral centre. | Experts Committee | Included in the sensitivity analysis (experts’ opinion) |
| DHC | Tests required at diagnosis (types and frequency) | Guidelines for the diagnosis and treatment of pulmonary hypertension published by the European Society of Cardiology (ESC) and the European Respiratory Society (ERS) [32] | Not included |
| DHC | Mean annual consumption of electrocardiogram, chest x-ray, biochemistry tests required at diagnosis per incident and prevalent patient  Mean annual consumption of CT angiogram required for patient management | Experts Committee | Included in the sensitivity analysis (experts’ opinion) |
| DHC | Number of visits, diagnostics | Experts Committee | Included in the sensitivity analysis (experts’ opinion) |
| DHC | Mean number of hospitalisation days | Literature [33] | Not included |
| DHC | As data on number of hospitalization days in FC I-II was aggregated in the original source, we assumed a distribution of 10% of patients in FC I and 90% in FC II in order for its aggregation | Experts Committee | Included in the sensitivity analysis (experts’ opinion) |
| DHC | Resource use related to transplants (number of transplants PAH year 2020) | Organización Nacional de Transplantes (National Transplant Organisation, ONT) [34] | Not included |
| DHC | We assumed that 47% of transplants were performed in FC III patients, and the remaining, in FC IV | Literature [35] | Not included |
| DHC | Resource consumption pharmacological treatment | Routine clinical practice (n=134 patients, n=255 drugs) | Not included |
| DHC | Doses pharmacological treatment | Routine clinical practice and product labels of each drug [36] | Not included |
| DHC | Administration costs pharmacological treatment | Literature [37] | Not included |
| DNHC | Supportive therapies, medical devices, and formal and informal care (consumption and frequency) | Survey: Spanish Pulmonary Hypertension Patient Organisation, HPE-ORG) [38] | Not included |
| DNHC | Supportive therapies: Patients use oxygen therapy on a daily basis (365 days per year) and that mechanical ventilation requires 12 days of hospital stay | Literature [39] | Not included |
| DNHC | Personal care was divided equally into formal and informal care whenever the type of assistance was left unspecified | Experts Committee | Included in the sensitivity analysis (experts’ opinion) |
| DNHC | % of free time from caregivers that are dedicated to informal care to patients, according to FC | Literature [40] | Not included |
| DNHC | Free time of each caregiver is approximately 42 hours per week (we excluded 8 daily hours of sleep and 10 daily hours work) | Experts Committee | Included in the sensitivity analysis (experts’ opinion) |
| IC | Loss of labour productivity by FC (early retirement, permanent disability, temporary leave or reduction of working hours) | Survey: Spanish Pulmonary Hypertension Patient Organisation, HPE-ORG) [38] | Included in the sensitivity analysis (key parameters) |
| IC | Work days lost to disability: a total of 29.6 days lost per year were applied to patients in FC I-II, and none to patients in FC III-IV | Number of days: literature [41]  Distribution by FC: Survey: Spanish Pulmonary Hypertension Patient Organisation, HPE-ORG) [38] | Not included |
| IC | Time spent on medical visits and tests: An average duration of 30 to 60 minutes was assumed for each visit/test, with an average travelling time of 1 hour (round trip). For hospitalisations and NIMV, the average duration considered was 8 hours per day (equivalent to one working day) | Experts Committee | Included in the sensitivity analysis (experts’ opinion) |
| Prevalent | For prevalent patients, follow-up medical visits and tests were considered, and no drug administration cost was assumed | Literature [37] | Not included |
| Incident | - Diagnosis tests and first medical visits were assumed, regardless of the patient’s FC - The cost of care management and supportive therapies were added, assuming that incident patients consumed half the annual resources of prevalent ones, as the exact date of diagnosis couldn’t be specified - Transplant costs were not included - Costs of early retirement, permanent disability, temporary leave, or reduction of working hours were not included for incident patients. | Literature [32, 42] | Not included |
| Epidemiology: prevalence and incidence | - A low and high prevalence range was applied (16.0 and 25.9, respectively) and an incidence of 3.7 to the adult Spanish population of 39.1 million in 2020. | Literature [43–46] | Included in the sensitivity analysis (experts’ opinion) |

**DHC**: Direct Healthcare Costs

**Scenarios for the sensitivity analysis**

In order to examine the model’s robustness, a deterministic sensitivity analysis was carried out, including twelve different scenarios based on the possible variation of the most sensitive parameters. The scenarios were validated by the experts committee. **Table (S2).2** describes those variations in detail.

**Table (S2).2: Scenarios for the sensitivity analysis**

| **#** | **Domain** | **Parameter** | **BC Source** | **SA rationale** | **Base Case** | **Lower case** | **Upper case** |
| --- | --- | --- | --- | --- | --- | --- | --- |
| 1 | Epidem. | Prevalence | Literature | Max prevalence, Europe, literature | Min (16.0 ppm) – Max (25.9 ppm) | - | 55 ppm [47] |
| 2 | DHC | Number of visits, diagnostics | Experts Opinion | ± 40%-50% | Primary care: 5  Cardiology: 2;  Pneumology:2 | Primary Care: -2  Others: -1 | Primary Care: +2  Others: +1 |
| 3 | DHC | Medical tests, diagnostics | Experts Opinion | ± 60% | Electrocardiogram Chest x-ray, Biochemistry (2.5 tests each) | -1.5 | +1.5 |
| 4 | DHC | Emergency visits | Experts Opinion | Extremes | 40% emergency service (ES)  60% unscheduled visits to referral (UV) | 0% ES  100% UV | 100% ES  0% UV |
| 5 | DHC | Distribution by FC, number of hospitalization days, FC I-II | Experts Opinion | Extremes | FC I: 10%  FC II: 90% | FC I: 100%  FC II: 0% | FC I: 0%  FC II: 100% |
| 6 | DHC | Discount on drug costs | Official data | From -43.1% to 0% | PVL (+) 4% VAT (–) RDL discount | -43.1% [48] | 0% |
| 7 | DHC / DNHC | Unit cost for tests, visits, transplant, transport, hospitalisation and support therapies | Official data | Extremes | Median from prices/unit costs in Autonomous Communities | Minimum unit costs in Autonomous Communities | Maximum unit cost in Autonomous Communities |
| 8 | DNHC | Number of hours dedicated by caregivers to patients, per week | Literature | ± 28%-33% | FC I-II: 21  FC III: 26  FC IV: 29 | FC I-II: 14  FC III: 18  FC IV: 21 | FC I-II: 27  FC III: 34  FC IV: 38 |
| 9 | DNHC | Distribution, formal and informal care | Literature | Formal: 17%-36% | Formal: 26%  Informal: 74% | Formal: 17% Informal: 83% | Formal: 36%  Informal: 64% |
| 10 | DNHC | Time spent on medical visits and tests | Experts Opinion | Visits/tests: ± 25%-50%  Travel: ± 50%  Hosp/NIMV: ±25% | Visit/test: 30-60m  Travel: 1h (round)  Hosp/NIMV: 8h/day | Visit/test: -15m  Travel: -30m  Hosp/NIMV: -2h | Visit/test: +15m Travel: +30m Hosp/NIMV: +2h |
| 11 | DNHC / IC | Wages / average working hours | Official data | ± 5% | INE | -5% | +5% |
| 12 | IC | Loss of labour productivity: (a) ER% + PL%; (b) TL% | Literature | ± 10-20% | FC I-II: 60/20  FC III: 100/0  FC IV: 100/0 | FC I-II: 40/30  FC III: 80/20  FC IV: 90/10 | FC I-II: 80/10  FC III/FC IV=BC |

**BC**: Base Case. **DHC**: Direct Healthcare Costs. **DNHC**: Direct Non-Healthcare Costs. **Epidem.**: Epidemiology. **ER**: Early Retirement. **ES**: Emergency Services. **FC**: Functional Class. **h**: hours. **Hosp**: Hospitalisations. **IC**: Indirect Costs. **INE**: National Statistics Institute. **LC**: Lower Case. **m**: minutes. **NIMV**: Non-Invasive Mechanical ventilation. **PL**: Permanent Leave. **RDL**: Royal Decree. **SA**: Sensitivity analysis **TL**: Temporary Leave. **UC**: Upper Case. **UV**: Unscheduled visits to referral, **VAT**: Value Added Tax.

**Results**

**Table (S2).3** and **Figure (S2).1** show the sensitivity analysis results. The most sensitive parameter was prevalence, with an impact of 105% (+ €111 million versus base case) over total costs estimated. Changes in unit costs could derive in -9% (€9 million) or +44% (€46 million). A drug discount could reduce total costs up to 22% (€23 million). The other parameters varied at a median of between -0.7% and +0.4% (ranging from -3% to +3%).

**Table (S2).3: Results of the sensitivity analysis, variations in € and % vs. base case, lower and upper case, lower-end of prevalence range and higher-end of prevalence range**

| **#** | **Description** | **Lower case (€)** | | **Lower Case (%)** | | **Upper Case (€)** | | **Upper case (%)** | |
| --- | --- | --- | --- | --- | --- | --- | --- | --- | --- |
|  |  | **LEPR** | **HEPR** | **LEPR** | **HEPR** | **LEPR** | **HEPR** | **LEPR** | **HEPR** |
| 1 | Prevalence | n.a. | n.a. | n.a. | n.a. | n.a. | 110,928,874 | n.a. | 104.52 |
| 2 | Visits, diagnostics | -40,783 | -40,783 | -0.06 | -0.04 | 40,783 | 40,783 | 0.06 | 0.04 |
| 3 | Tests, diagnostics | -26,993 | -26,993 | -0.04 | -0.03 | 26,993 | 26,993 | 0.04 | 0.03 |
| 4 | Emergency visits | -163,250 | -253,792 | -0.24 | -0.24 | 244,875 | 380,687 | 0.36 | 0.36 |
| 5 | Hospitalisations, FC I-II | -561,854 | -873,471 | -0.83 | -0.83 | 62,428 | 97,052 | 0.09 | 0.09 |
| 6 | Drug discount | -14,882,649 | -23,106,662 | -21.92 | -21.77 | n.a. | n.a. | n.a. | n.a. |
| 7 | Unit costs | -5,881,793 | -8,994,413 | -8.66 | -8.47 | 30,007,395 | 45,702,464 | 44.20 | 43.06 |
| 8 | Hours caregiver | -2,167,599 | -3,369,797 | -3.19 | -3.18 | 2,146,677 | 3,337,271 | 3.16 | 3.14 |
| 9 | Formal / informal care | -68,578 | -106,613 | -0.10 | -0.10 | 69,815 | 108,536 | 0.10 | 0.10 |
| 10 | Time spent visits, etc. | -486,540 | -756,386 | -0.72 | -0.71 | 486,540 | 756,386 | 0.72 | 0.71 |
| 11 | Wages / working hours | -1,858,290 | -2,968,866 | -2.74 | -2.80 | 1,931,547 | 3,086,858 | 2.85 | 2.91 |
| 12 | Loss labour productivity | -1,000,533 | -1,619,613 | -1.47 | -1.53 | 597,156 | 966,646 | 0.88 | 0.91 |

**HEPR**: higher-end prevalence range. **LEPR**: lower-end prevalence range

**Figure (S2).1: Tornado diagram, % of variation in total costs vs. base case**


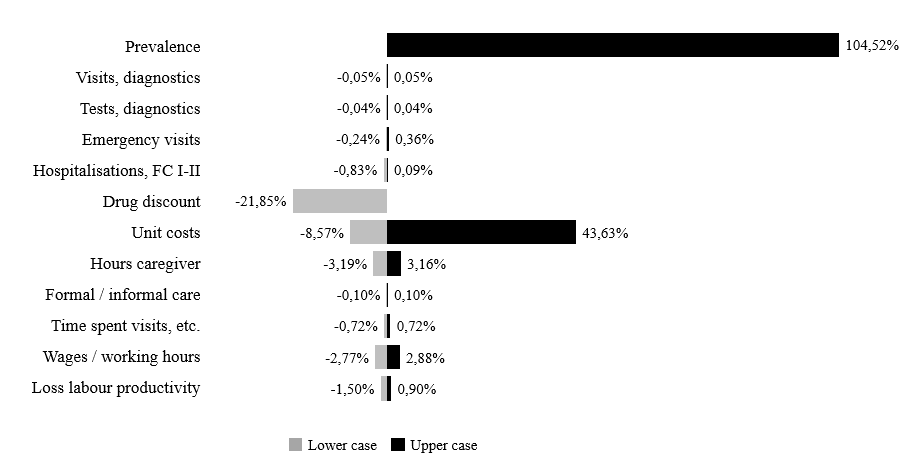


# **References**

1. Boletín Oficial de Aragón - BOA, Núm. 156 (10/08/2012). Resolución de 30 de julio de 2012, de la Dirección Gerencia del Servicio Aragonés de Salud, sobre revisión de las tarifas a aplicar por la prestación de servicios sanitarios a terceros obligados al pago o a usuarios sin derecho a asistencia sanitaria en la Comunidad Autónoma de Aragón.

2. Boletín Oficial del Principado de Asturias, Núm. 219 (13/11/2019). Decreto 194/2019, de 31 de octubre, de segunda modificación del Decreto 87/2009, de 29 de julio, por el que se establecen los precios públicos a aplicar por el Servicio de Salud del Principado de Asturias por la prestación de servicios sanitarios.

3. Boletín Oficial de las Islas Baleares - BOIB, Núm. 128 (16/10/2018). Núm. 10517. Resolución del director general del Servicio de Salud de las Islas Baleares de corrección de errores de la Resolución del director general del Servicio de Salud de las Islas Baleares de 29 de diciembre de 2017 por la que se modifica la Orden de la consejera de Salud y Consumo de 22 de diciembre de 2006 por la que se establecen los precios públicos a aplicar por los centros sanitarios de la Red Pública de las Illes Balears por la prestación de servicios sanitarios, cuando existan terceros obligados al pago o usuarios sin derecho a asistencia sanitaria de la Seguridad Social.

4. Boletín Oficial de Canarias - BOC, Núm. 67 (5/4/2017) - 1616 Servicio Canario de la Salud.- Resolución de 29 de marzo de 2017, del Director, por la que se modifica la cuantía de los precios públicos de servicios sanitarios previstos en el Decreto 81/2009, de 16 de junio, por el que se establecen los precios públicos de los servicios sanitarios prestados por el Servicio Canario de la Salud y se fijan sus cuantías. http://www.gobiernodecanarias.org/boc/2017/067/002.html. Accessed 20 Jun 2021.

5. Boletín Oficial de la Junta de Andalucía - BOJA, Núm. 92 (15/5/2018) Orden de 8 de mayo de 2018, por la que se modifica la Orden de 14 de octubre de 2005, por la que se fijan los precios públicos de los servicios sanitarios prestados por centros dependientes del Sistema Sanitario Público de Andalucía.

6. Boletín Oficial de Cantabria - BOC Núm. 248 (29/12/2017) - Orden SAN/35/2017, de 15 de diciembre, por la que se fi jan las cuantías de los Precios Públicos de los Servicios Sanitarios prestados por el Servicio Cántabro de Salud.

7. Diario Oficial de Extremadura - DOE, Núm. 32 (17/2/2021) - RESOLUCIÓN de 10 de febrero de 2021, de la Vicepresidenta Primera y Consejera, por la que se publican las tarifas actualizadas de las tasas y precios públicos de la Comunidad Autónoma de Extremadura en virtud de lo dispuesto en la Ley de Presupuestos Generales de la Comunidad Autónoma de Extremadura para 2021.

8. Normativa vigente. Tasas y precios de la Comunidad Autónoma - Diario Oficial de Galicia - DOG Núm. 240 (11/12/2003) - Ley 6/2003, de 9 de diciembre, de tasas, precios y exacciones Reguladoras de la comunidad autónoma de galicia. 2020.

9. Boletín Oficial de la Comunidad de Madrid – BOCM, Núm. 198 (21/8/2017) - Orden 727/2017, de 7 de agosto, del Consejero de Sanidad, por la que se fijan los precios públicos por la prestación de los servicios y actividades de naturaleza sanitaria de la red de centros de la Comunidad de Madrid.

10. Boletín Oficial de Castilla y León - BOCYL, Núm. 249 (30/12/2013) - Decreto 83/2013, de 26 de diciembre, por el que se actualizan los precios públicos de la Comunidad de Castilla y León y la tasa por actuaciones administrativas relativas a actividades agrícolas.

11. Diario Oficial de Castilla-La Mancha - DOCM, Núm. 226 (21/11/2014) - Orden de 17/11/2014, de la Consejería de Sanidad y Asuntos Sociales, por la que se establecen los precios públicos de la asistencia sanitaria y de los servicios prestados en la red de centros sanitarios dependientes del Servicio de Salud de Castilla-La Mancha.

12. Diari Oficial de la Generalitat de Catalunya - DOGC, Núm. 8153 - (12/6/2020) - Orden SLT/71/2020, de 2 de junio, por la que se regulan los supuestos y conceptos facturables y se aprueban los precios públicos correspondientes a los servicios que presta el Instituto Catalán de la Salud. https://infoboe-production.s3.eu-central-1.amazonaws.com/documents/fc27d9c2c6b9b9a029b20ebf5d6613d4-20200612.pdf. Accessed 20 Jun 2021.

13. Boletín Oficial de la Región de Murcia - BORM, Núm. 133 (11/6/2020) - Orden de 29 de mayo de 2020 de la Consejería de Presidencia y Hacienda, por la que se publican las tarifas de las tasas y precios públicos aplicables en el año 2020.

14. Boletín Oficial de Navarra - BON, Núm. 14 (22/1/2019) - Resolución 1564/2018, de 20 de diciembre, del Director Gerente del Servicio Navarro de Salud-Osasunbidea, por la que se establecen las tarifas por los servicios prestados por el Servicio Navarro de Salud-Osasunbidea. http://bon.navarra.es/es/anuncio/-/texto/2019/14/5/. Accessed 20 Jun 2021.

15. Tarifas para facturación de servicios sanitarios y docentes de Osakidetza para el año 2020 (12/2019) - Acuerdo de 19 de diciembre de 2019, del consejo de administración del ente público Osakidetza, por el que se aprueban las tarifas por prestación de servicios sanitarios y docentes a terceros obligados al pago durante el ejercicio 2020.

16. Boletín Oficial del Estado - BOE Ceuta y Melilla, Núm. 180 (29/7/2013) Sec. I. Pág. 55225 - Resolución de 19 de julio de 2013, del Instituto Nacional de Gestión Sanitaria, sobre revisión de precios a aplicar por los centros sanitarios del Instituto Nacional de Gestión Sanitaria en Ceuta y Melilla, por las asistencias prestadas en los supuestos cuyo importe ha de reclamarse a los terceros obligados al pago o a los usuarios sin derecho a la asistencia sanitaria de la Seguridad Social, así como por los servicios prestados por el Centro Nacional de Dosimetría y por la reproducción de documentos de la biblioteca de la entidad gestora.

17. Boletín Oficial de la Junta de Andalucía - BOJA, Núm. 210 (27/10/2005) - Orden de 14 de octubre de 2005, por la que se fijan los precios públicos de los servicios sanitarios prestados por Centros dependientes del Sistema Sanitario Público de Andalucía. Servicio Andaluz de Salud. https://www.sspa.juntadeandalucia.es/servicioandaluzdesalud/sites/default/files/sincfiles/wsas-media-sas_normativa_mediafile/2019/orden2005_precios_publicos.pdf. Accessed 20 Jun 2021.

18. Diario Oficial de la Generalitat Valenciana - DOGV, Núm. 8202 (30/10/2017) - Ley 20/2017, de 28 de diciembre, de la Generalitat, de tasas. [2017/12159]. 2017. https://dogv.gva.es/portal/ficha_disposicion.jsp?L=1&sig=011728%2F2017. Accessed 20 Jun 2021.

19. Diario Oficial de Extremadura - DOE, Núm. 28 (11/2/2020) - Resolución de 6 de febrero de 2020, de la Vicepresidenta Primera y Consejera, por la que se publican las tarifas actualizadas de las tasas y precios públicos de la Comunidad Autónoma de Extremadura en virtud de lo dispuesto en la Ley de Presupuestos Generales de la Comunidad Autónoma de Extremadura para 2020. (2020060240).

20. Prestación de los servicios y actividades de naturaleza sanitaria de la Red de Centros de la Comunidad de Madrid. Anexo - Precios públicos por servicios y actividades.

21. Boletín Oficial de La Rioja - BOR, Núm. 156 (19/12/2014) Pág. 24921 - Orden 12/2014, de 16 de noviembre de 2014, de la Consejería de Administración Pública y Hacienda por la que se establece y regula el precio público por los servicios sanitarios prestados a particulares en los centros del Servicio Riojano de Salud.

22. Boletín Oficial de Aragón - BOA, Núm. 135 (12/07/2011) - Resolución de 1 de enero de 2011, de la Directora-Gerente del Consorcio de Salud, por la que se aprueban las tarifas a aplicar en el seno del Consorcio a los terceros obligados al pago o a los usuarios sin derecho a la asistencia sanitaria de la Seguridad Social, con excepción de aquellos supuestos cuyas tarifas vengan reguladas por convenios, contratos o conciertos específicos.

23. Boletín Oficial de la Junta de Andalucía - BOJA, Núm. 210 (21/10/2015) Pág. 46 - Orden de 14 de octubre de 2005, por la que se fijan los precios públicos de los servicios sanitarios prestados por Centros dependientes del Sistema Sanitario Público de Andalucía.

24. Boletín Oficial de Aragón - BOA, Núm. 165 (29/08/2017) - Orden SAN/1221/2017, de 21 de julio, por la que se establecen los precios y tarifas máximas aplicables en la prestación de servicios sanitarios con medios ajenos al Sistema de Salud de Aragón. 2017. http://www.boa.aragon.es/cgi-bin/EBOA/BRSCGI?CMD=VEROBJ&MLKOB=977342223030. Accessed 20 Jun 2021.

25. Boletín Oficial de las Islas Baleares - BOIB, Núm. 002 (4/1/2018) - Resolución del director general del Servicio de Salud de las Islas Baleares por la que se modifica la Orden de la consejera de Salud y Consumo de 22 de diciembre de 2006 por la que se establecen los precios públicos a aplicar por los centros sanitarios de la Red Pública de las Islas Baleares por la prestación de servicios sanitarios, cuando existan terceros obligados al pago o usuarios sin derecho a asistencia sanitaria de la Seguridad Social. http://www.caib.es/eboibfront/es/2018/10758/seccion-iii-otras-disposiciones-y-actos-administra/472. Accessed 21 Jun 2021.

26. Boletín Oficial del Principado de Asturias, Núm. 77 (4/4/2013) - Resolución de 25 de febrero de 2013, de la Consejería de Hacienda y Sector Público, por la que hace pública la relación de las cuantías exigibles por tasas y precios públicos en el ejercicio 2013. 2013. https://sede.asturias.es/bopa/2013/10/03/2013-18043.pdf. Accessed 20 Jun 2021.

27. Instituto Nacional de Estadística (INE). INEbase / Nivel y condiciones de vida (IPC) /Índices de precios de consumo y vivienda /Índice de precios de consumo 2020. INE. https://www.ine.es/dyngs/INEbase/es/operacion.htm?c=Estadistica_C&cid=1254736176802&menu=ultiDatos&idp=1254735976607. Accessed 19 Aug 2021.

28. Ministerio de Sanidad, Consumo y Bienestar Social - Portal Estadístico del SNS - Registro de Altas de los Hospitales Generales del Sistema Nacional de Salud. CMBD. Norma Estatal. https://www.mscbs.gob.es/estadEstudios/estadisticas/cmbd.htm. Accessed 21 Jun 2021.

29. Consejo General de Colegios Oficiales de Farmacéuticos. Base de datos de información sanitaria (BOT PLUS). 2021. https://botplusweb.portalfarma.com/.

30. Instituto Nacional de Estadística - INE. Número medio de horas efectivas semanales trabajadas por todos los ocupados (hayan o no trabajado en la semana) por situación profesional, sexo y rama de actividad (empleo principal) (4880). INE. https://www.ine.es/jaxiT3/Tabla.htm?t=4880&L=0. Accessed 21 Jun 2021.

31. Instituto Nacional de Estadística - INE. Sexo y grupos principales de ocupación (28186). INE. https://www.ine.es/jaxiT3/Tabla.htm?t=28186&L=0. Accessed 21 Jun 2021.

32. Galiè N, Humbert M, Vachiery J-L, Gibbs S, Lang I, Torbicki A, et al. ESC/ERS Guidelines for the diagnosis and treatment of pulmonary hypertension: The Joint Task Force for the Diagnosis and Treatment of Pulmonary Hypertension of the European Society of Cardiology (ESC) and the European Respiratory Society (ERS): Endorsed by: Association for European Paediatric and Congenital Cardiology (AEPC), International Society for Heart and Lung Transplantation (ISHLT). Eur Respir J. 2015;46:903–75.

33. Dufour R, Pruett J, Hu N, Lickert C, Stemkowski S, Tsang Y, et al. Healthcare resource utilization and costs for patients with pulmonary arterial hypertension: real-world documentation of functional class. J Med Econ. 2017;20:1178–86.

34. Organización Nacional de Trasplantes - ONT, Memoria actividad donación y trasplante pulmonar. España 2020.

35. López-Meseguer M, Román A, Monforte V, Bravo C, Solé J, Morell F. Trasplante bipulmonar en hipertensión pulmonar. Una serie de 15 pacientes. Arch Bronconeumol. 2009;45:366–70.

36. AEMPS. Centro de información de medicamentos (CIMA). 2018. https://www.aemps.gob.es/cima/publico/home.html. Accessed 18 Oct 2018.

37. Roman A, Barberà JA, Escribano P, Sala ML, Febrer L, Oyagüez I, et al. Cost effectiveness of prostacyclins in pulmonary arterial hypertension. Appl Health Econ Health Policy. 2012;10:175–88.

38. La carga socioeconómica de la hipertensión pulmonar en España. Hipertensión Pulmonar. https://hipertension-pulmonar.com/la-asociacion/estudios/255-la-carga-socioeconomica-de-la-hipertension-pulmonar-en-espana. Accessed 28 Jun 2021.

39. Hawn JM, Bauer SR, Wanek MR, Li M, Wang X, Duggal A, et al. Effectiveness, Safety, and Economic Comparison of Inhaled Epoprostenol Brands, Flolan and Veletri, in Acute Respiratory Distress Syndrome. Ann Pharmacother. 2020;54:434–41.

40. PHA Europe. The impact of pulmonary arterial hypertension (PAH) on the lives of patients and carers: results from an international survey. European Pulmonary Hypertension Association. PHA Europe. 2012. https://www.phaeurope.org/wp-content/uploads/PAH_Survey_FINAL.pdf. Accessed 3 Feb 2021.

41. Joish VN, Kreilick C, Germino R, Muccino D. Estimation Of Pulmonary Arterial Hypertension On Productivity Losses In The United States. Value Health. 2014;17:A118.

42. Ministerio de Inclusión, Seguridad Social y Migraciones. Seguridad Social: Trámites y Gestiones. https://www.seg-social.es/wps/portal/wss/internet/InformacionUtil/44539. Accessed 26 Jan 2022.

43. Escribano-Subias P, Blanco I, Lopez-Meseguer M, Jiménez C, Román A, Castillo-Palma M, et al. REHAP investigators. Survival in pulmonary hypertension in Spain: Insights from the Spanish registry. Eur Respir J Off J Eur Soc Clin Respir Physiol. 2012;40:596–603.

44. Hoeper MM, Huscher D, Pittrow D. Incidence and prevalence of pulmonary arterial hypertension in Germany. Int J Cardiol. 2016;203:612–3.

45. Escribano-Subias P, Blanco I, López-Meseguer M, Lopez-Guarch CJ, Roman A, Morales P, et al. Survival in pulmonary hypertension in Spain: insights from the Spanish registry. Eur Respir J. 2012;40:596–603.

46. Última Nota de prensa. Cifras de Población. Provisionales a 1 de julio de 2020. Instituto Nacional de Estadística (INE). https://www.ine.es/dyngs/INEbase/es/operacion.htm?c=Estadistica_C&cid=1254736176951&menu=ultiDatos&idp=1254735572981.

47. Leber L, Beaudet A, Muller A. Epidemiology of pulmonary arterial hypertension and chronic thromboembolic pulmonary hypertension: identification of the most accurate estimates from a systematic literature review. Pulm Circ. 2021;11:2045894020977300.

48. DiarioFarma. Precios notificados: cada año hay más y con mayor diferencia sobre el real. diariofarma. 2019. https://www.diariofarma.com/2019/02/12/precios-notificados-cada-ano-hay-mas-y-con-mayor-diferencia-sobre-el-real.
